# Subcellular localization of MCM2 correlates with the prognosis of ovarian clear cell carcinoma

**DOI:** 10.18632/oncotarget.25613

**Published:** 2018-06-15

**Authors:** Gulinisha Aihemaiti, Morito Kurata, Daichi Nogawa, Akiko Yamamoto, Tatsunori Mineo, Iichiroh Onishi, Yuko Kinowaki, Xiao-Hai Jin, Anna Tatsuzawa, Naoyuki Miyasaka, Masanobu Kitagawa, Kouhei Yamamoto

**Affiliations:** ^1^ Department of Comprehensive Pathology, Graduate School of Medical and Dental Sciences, Tokyo Medical and Dental University, Tokyo 113–8510, Japan; ^2^ Department of Analytical Information of Clinical Laboratory Medicine, Graduate School of Health Care Science, Bunkyo Gakuin University, Tokyo 113–8668, Japan; ^3^ Department of Obstetrics and Gynecology, Graduate School of Medical and Dental Sciences, Tokyo Medical and Dental University, Tokyo 113–8510, Japan

**Keywords:** ovarian cancer, pathology, clinicopathologic study, MCM2, cancer therapy target

## Abstract

Highly malignant tumors overexpress the minichromosome maintenance 2 (MCM2) protein in the nucleus, which is associated with advanced tumor grade, advanced stage, and poor prognosis. In this study, we showed that MCM2 is highly expressed in clinical samples of ovarian clear cell carcinoma. Although MCM2 expression was mainly localized to the nuclei as in other cancers, a few cases exhibited cytoplasmic localization of MCM2. Surprisingly, tumor samples with cytoplasmic MCM2 demonstrated excellent prognosis, showing 100% survival during the observation period of more than 200 months. However, cases with nuclear expression of MCM2 exhibited approximately 78% 5-year-survival rate. In a previous study, we showed that Friend leukemia virus (FLV) envelope protein gp70 bound to MCM2, impaired its nuclear translocation, and enhanced DNA damage-induced apoptosis in FLV-infected hematopoietic cells with high levels of MCM2. As expected, clear cell carcinoma cells with cytoplasmic expression of MCM2 exhibited significantly higher apoptotic cell ratio than that of cells with nuclear MCM2 expression. *In vitro* experiments using ovarian cancer cells with cytoplasmic expression of MCM2 demonstrated that transfection of MCM2-ΔN enhanced DNA damage-induced apoptosis. Therefore, cytoplasmic localization of MCM2 significantly correlated with increased apoptosis in clear cell carcinoma cells, resulting in improved prognosis.

## INTRODUCTION

Ovarian cancer is a heterogeneous disease with histological subtypes that have different biological characteristics and prognoses [1, 2]. Among the subtypes, carcinoma with clear cell histology, called clear cell carcinoma, is relatively common in Japan. It was first defined as a histological subtype in 1973 by the World Health Organization (WHO) [3] and found to be distinct from other epithelial ovarian carcinomas [4] with frequent mutations of *ARID1A* and *PIK3CA* [2]. The origin of clear cell carcinoma is associated with endometriosis or clear cell adenofibroma. In contrast, serous cyst adenocarcinoma likely arises from Mullerian epithelium, which derives from either the ovarian surface epithelium or fallopian tube [1]. Importantly, clear cell carcinoma of the ovary is resistant to platinum-based conventional chemotherapy and exhibits worse prognosis than the more common serous adenocarcinoma or ovarian endometrioid adenocarcinoma [4–9]. Therefore, a more precise understanding of the basic biology is needed for establishing a specific therapeutic strategy against clear cell carcinoma, which is highly distinct from other common ovarian carcinomas.

Minichromosome maintenance (MCM) 2 is one of six related proteins that comprise the MCM complex (MCM2-7), which has an essential role in DNA replication [10]. Previous studies using human samples have established MCM2 as a proliferation marker of cancer cells. High expression MCM2 level in malignant tumors, including ovarian cancer, is associated with several clinico-pathological parameters such as advanced tumor grade, advanced stage, and poor prognosis [11–16]. We previously demonstrated that Friend leukemia virus (FLV) infection markedly enhanced DNA damage-induced apoptosis in hematopoietic cells in C3H mice via activation of ATM, DNA-PK, and p53 [17]. The increased apoptosis rate was almost exclusively observed in C3H mice [18] expressing high levels of MCM2 in hematopoietic cells. We also demonstrated that the FLV-derived envelope protein gp70 enhanced cellular apoptotic signaling specifically in cells that overexpressed MCM2 [19]. gp70 bound directly to the nuclear localization signal of MCM2 and inhibited its nuclear translocation. The cytoplasmic MCM2-gp70-complex bound to protein phosphatase 2A (PP2A), interfered with the PP2A-DNA-PK interaction, and enhanced DNA damage-induced apoptosis via the activation of p53 by DNA-PK [20]. These results suggest that regulation of the molecular dynamics of MCM2 by gp70 offers a novel therapeutic approach against malignant tumors that express high levels of MCM2. To develop an MCM2-targeted therapy, we proposed a method for efficiently introducing the gp70 protein into cancer cells [15].

In the present study, we analyzed the expression of MCM2 in human ovarian cancer. We demonstrated cytoplasmic expression of MCM2 in a subset of clear cell carcinoma cases. However, MCM2 localized to the nuclei in other types of ovarian carcinomas such as serous carcinoma and endometrioid carcinoma. Therefore, immunohistochemical localization studies of MCM2 were performed in association with cellular apoptosis in clear cell carcinoma. Next, we examined the correlation between subcellular localization of MCM2 and clinico-pathological features, and prognostic factors of patients with ovarian clear cell carcinoma. Furthermore, we introduced *MCM2-*Δ*N*, which lacks the nuclear localization signal (NLS) domain, into human ovarian cancer cells and investigated whether cytoplasmic localization of the MCM2 protein had apoptosis-enhancing effects in human ovarian cancer.

## RESULTS

### Immunohistochemical analysis of MCM2 expression in ovarian carcinoma

**Figure 1:**
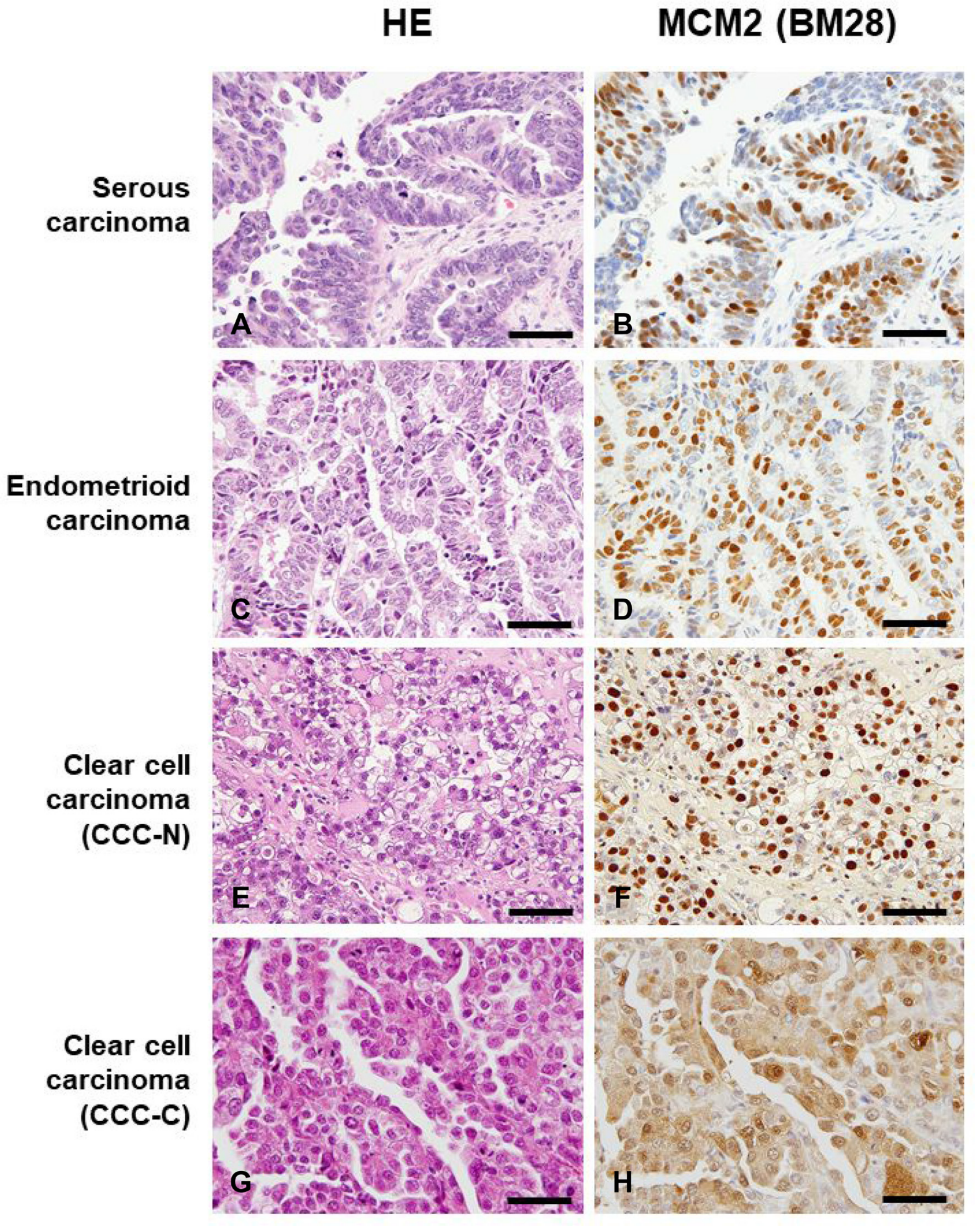
Histological features by hematoxylin and eosin (H&E) staining (**A**, **C**, **E**, **G**) and the immunohistochemical expression patterns of minichromosome maintenance 2 (MCM2; (**B**, **D**, **F**, **H**) in serous carcinoma, endometrioid carcinoma, and clear cell carcinoma using the monoclonal antibody BM28. Magnification of all images shown is ×400, and scale bar indicates 50 μm. Although MCM2 was primarily localized to the nuclei of cancer cells (B, D, F), a subset of clear cell carcinoma cases exhibited cytoplasmic MCM2 expression in addition to nuclear staining (H). Clear cell carcinoma with nuclear expression of MCM2 is designated as CCC-N, whereas clear cell carcinoma showing cytoplasmic/nuclear localization of MCM2 is indicated as CCC-C.

To determine whether MCM2 was expressed in ovarian carcinoma cells, immunohistochemical staining for MCM2 was performed in serous carcinoma, endometrioid carcinoma, and clear cell carcinoma using FFPE samples. The characteristics of ovarian cancer cases studied here are summarized in Table 1. Figure 1 shows the hematoxylin and eosin (H&E) staining features (Figure 1A, 1C, 1E, 1G) and expression patterns of MCM2 in serous carcinoma, endometrioid carcinoma, and clear cell carcinoma using the monoclonal antibody BM28. The staining patterns for MCM2 expression observed using the BM28 antibody were very similar to those observed using the N19 antibody (Supplementary Figure 1). The MCM2 staining results demonstrated that many of the ovarian carcinoma cells were positive for MCM2. Although MCM2 was mostly localized to the nuclei of cancer cells (Figure 1B, 1D, 1F), a subset of clear cell carcinoma samples exhibited cytoplasmic localization of MCM2 in addition to nuclear staining (Figure 1H, Supplementary Figure 1). The frequency of MCM2-positive cells in ovarian carcinomas is shown in Table 2. The MCM2-positive cell ratio was significantly higher in clear cell carcinoma with cytoplasmic/nuclear staining (CCC-C) than in serous carcinoma, endometrioid carcinoma, and clear cell carcinoma with nuclear staining (CCC-N) (*P* < 0.01, *P* < 0.01, *P* < 0.05, respectively). MCM2 is a histological proliferation marker with Ki-67. The Ki-67-positive cell ratio in CCC-C cases was significantly higher than that in CCC-N cases (Supplementary Figure 2A). However, the correlation between MCM2 (BM28) and Ki-67 labeling indices was not statistically significant in both CCC-N (*R* = −0.3322) and CCC-C (*R* = −0.0046) (Supplementary Figure 2).

**Table 1:** Patient characteristics in the present study

	Serous carcinoma (*n* = 60)	Endometrioid carcinoma (*n* = 37)	Clear cell carcinoma CCC-N (*n* = 55)	Clear cell carcinoma CCC-C (*n* = 22)
Age				
Median (Minimum-Max)	60 (38–79)	48 (30–76)	55 (38–87)	56 (39–77)
FIGO classification				
I	8	25	33	15
IA + IB	(3)	(12)	(15)	(5)
IC	(5)	(13)	(18)	(10)
II	4	4	6	5
III	35	6	12	1
IV	13	2	2	1
Lymph node metastasis				
Positive	13	3	6	1
Negative	10	18	33	18
Uncertain	37	16	16	3
Chemotherapy				
PTX + CBDCA	38	20	28	10
MMC + ETP + CDDP	0	0	5	5
PTX + CBDCA / CPT + CDDP	2	1	2	0
PTX + DTX + CBDCA	0	0	2	1
PTX + CBDCA + Bmab	0	0	1	1
Others	13	3	4	0
None	4	12	0	0
Uncertain	3	1	13	5

FIGO, International Federation of Gynecology and Obstetrics; PTX, paclitaxel; CBDCA, carboplatin; MMC, mitomycin-C; ETP, etoposide; CDDP, cisplatin; DTX, docetaxel; Bmab: bevacizumab.

**Table 2:** Frequency of MCM2 positive cells in ovarian carcinoma

Subtype	Number of cases	MCM2 positive cell ratio (%, mean ± 2SD)
Serous carcinoma	60	61.5 ± 17.47^*^
Endometrioid carcinoma	37	64.5 ± 13.57^†^
Clear cell carcinoma		
CCC-N	55	63.9 ± 12.90 ^‡^
CCC-C	22	71.4 ± 8.72^*,†,‡^

^*,†^*P* < 0.01, ^‡^*P* < 0.05

^*,†,‡^*P* values were calculated using Student's *t*-test.

### Subcellular localization of MCM2 and clinico-pathological parameters of clear cell carcinoma

**Figure 2:**
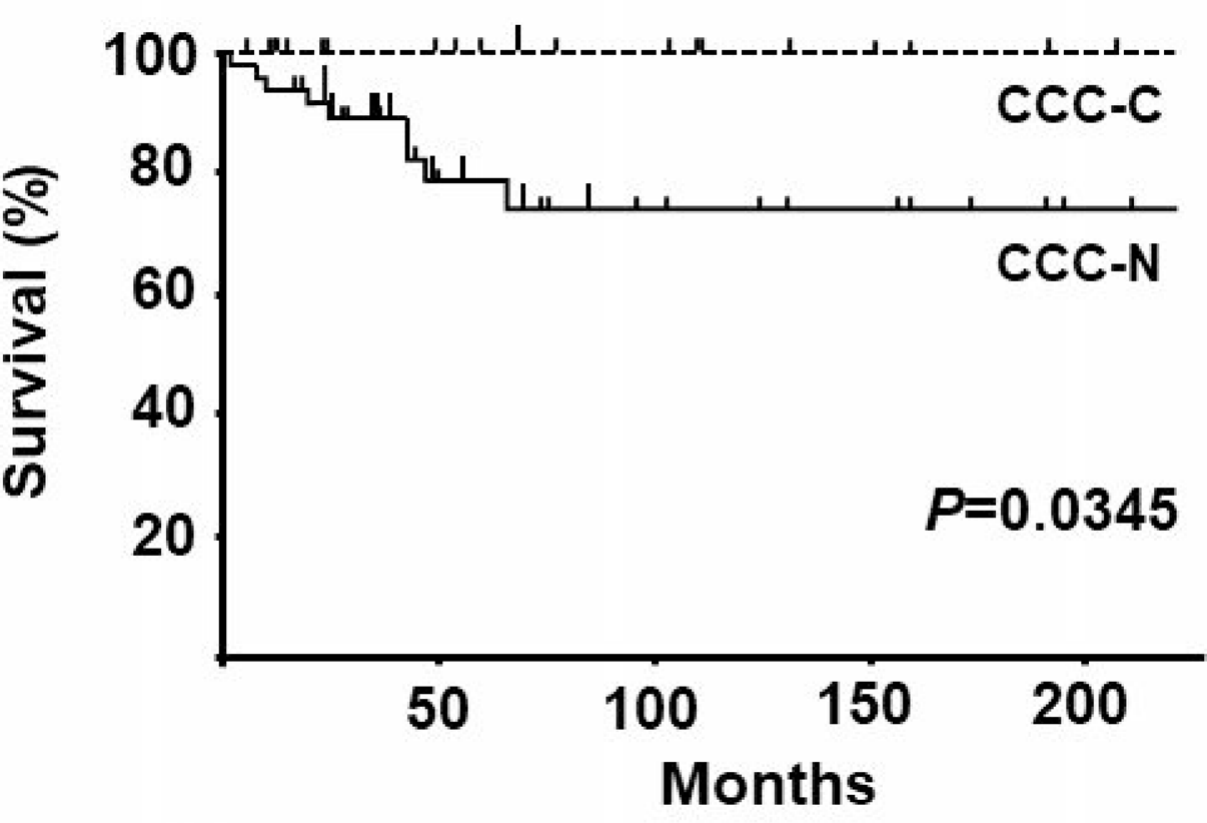
Overall survival curves for clear cell carcinoma with nuclear expression of MCM2 (CCC-N) and clear cell carcinoma with cytoplasmic/nuclear expression of MCM2 CCC-C cases As indicated, CCC-C cases exhibited a 100% 5-year survival rate, whereas CCC-N cases had a 5-year survival rate of 78%. Kaplan-Meier estimation of survival curves revealed significant differences in overall survival between CCC-N and CCC-C cases (*P* < 0.05).

As shown in Table 3, subcellular localization of MCM2 in clear cell carcinoma did not significantly correlate with clinico-pathological factors such as age, FIGO stage, TNM stage, tumor size, lymph node metastasis, and recurrence. As shown in Table 1, the majority of patients in this study were treated with platinum-based chemotherapy after surgical resection of tumors. The prognostic outcomes of patients with clear cell carcinoma were divided into 3 categories: 1) no evidence of disease (NED), 2) alive with disease (AWD), and 3) dead of disease (DOD) groups. CCC-C cases exhibited significantly better sensitivity against chemotherapy than CCC-N cases did (Table 3). The analysis of stage I patients revealed no significant correlation with the prognostic outcome although, in the DOD and AWD groups, CCC-N cases were 23.8% (5/21) and CCC-C cases were 13.3% (2/15) (Table 3). The overall survival curves of CCC-N and CCC-C cases are shown in Figure 2. As indicated, CCC-C cases exhibited a 100% 5-year-survival rate, whereas CCC-N cases had a 5-year-survival rate of 78%. Kaplan-Meier estimation of survival curves revealed significant differences in overall survival between CCC-N and CCC-C cases (*P* < 0.05).

**Table 3:** Patient characteristics as stratified by MCM2 localization status

	Clear cell carcinoma CCC-N	Clear cell carcinoma CCC-C	*P* value
Age			
≥60y	15	8	0.7943
<60y	32	15	
FIGO stage			
I, II	30	20	0.1164
III, IV	11	2	
Tumor size			
<4 cm	10	2	0.3125
≥4 cm	37	21	
TNM stage			
pT1, 2	31	18	0.1482
pT3, 4	10	1	
Lymph node metastasis			
Positive	6	1	0.4075
Negative	33	18	
Recurrence			
Recurrence	10	2	0.1883
No recurrence	31	20	
Chemosensitivity			
NED	22	15	0.0413
AWD+DOD (total number)	9+10 (19)	3+0 (3)	
Chemosensitivity (stage I)			
NED	16	13	0.6738
AWD+DOD (total number)	1+4 (5)	2+0 (2)	
Chemosensitivity (stage IC)			
NED	9	9	0.3408
AWD+DOD (total number)	1+4 (5)	1+0 (1)	

FIGO, International Federation of Gynecology and Obstetrics.

NED, no evidence of disease.

AWD, alive with disease.

DOD, dead of disease.

*P* values were calculated using χ^2^-test.

### Univariate and multivariate analyses of the subcellular localization of MCM2 and other factors influencing overall survival

Table 4 shows the results of the univariate and multivariate analyses for age, FIGO stage, TNM stage, tumor size, lymph node metastasis, recurrence, and MCM2 localization. Among these factors, lymph node metastasis, recurrence, and MCM2 localization correlated with overall survival, as determined by univariate analysis. Furthermore, recurrence and MCM2 localization were independent factors influencing the overall survival of clear cell carcinoma cases, as determined by multivariate analysis.

**Table 4:** Univariate and multivariate analyses of MCM2 localization influencing overall survival of cases with clear cell carcinoma

Variable	Category	No. of patients	*P* value by Wilcoxon test	HR^1)^	95% Cl^2)^	*P* value by Cox proportional hazards
Age	<60y	47	0.0741	17.5782		0.0067
	≥60y	23				
FIGO stage	I, II	47	0.1200			-^3)^
	III, IV	13				
TNM stage	pT1, 2	49	0.2903			-
	pT3, 4	11				
Tumor size	<4cm	12	0.7809			-
	≥4cm	58				
Lymph node metastasis	Positive	7	0.0090			0.0804
	Negative	51				
Recurrence	Recurrence	12	0.0100	6.7451	1.1609–39.19	0.0335
	No recurrence	51				
MCM2 localization	Nucleus	47	0.0345	0.0492	0.0049–0.4901	0.0102
	Cytoplasm	22				

^1)^HR, Hazard ratio; ^2)^CI, Confidence Interval; ^3)^-, Confounding factor.

### Apoptotic cell ratio of clear cell carcinoma cells

**Figure 3:**
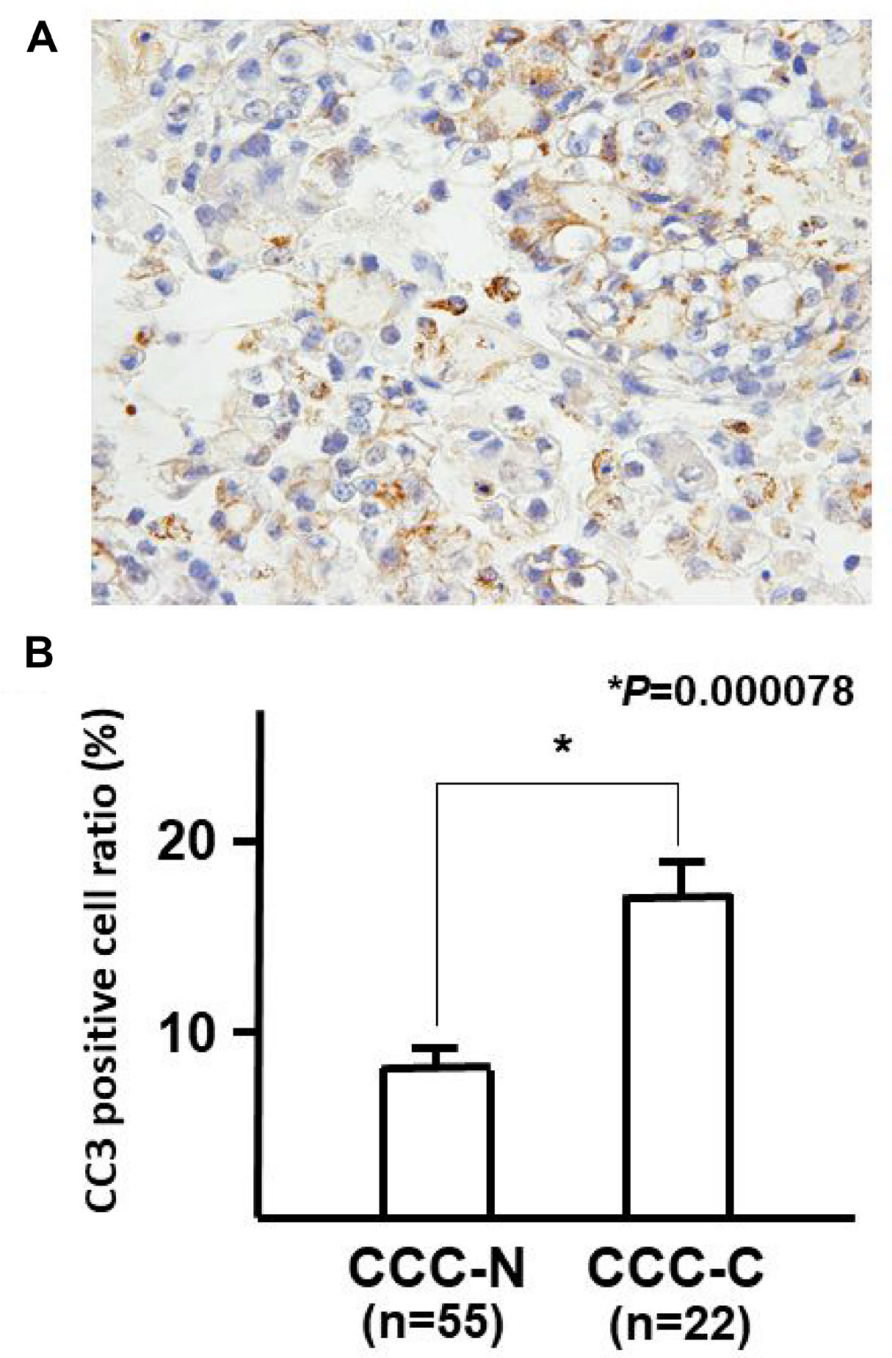
Immunohistochemical staining for CC3 (**A**) and the frequency of CC3-positive cells in clear cell carcinoma with nuclear expression of MCM2 (CCC-N; *n* = 55) and clear cell carcinoma with cytoplasmic/nuclear expression of MCM2 (CCC-C; *n* = 22) cases (**B**). CC3 was frequently positive in the nuclei of CCC-C cases. The frequency of CC3-positive cells in CCC-C was significantly higher than that in CCC-N (B, *P* < 0.01). *P* values were calculated using Student's *t*-test

Since cytoplasmic localization of MCM2 enhanced DNA damage-induced apoptosis in our previous experiments using an animal model [20], we determined the apoptotic cell ratio in CCC-N and CCC-C cases by demonstrating the immunohistochemical expression of CC3. As shown in Figure 3A, CC3 was frequently positive in the nuclei of CCC-C cases. The frequency of CC3-positive cells in CCC-C was significantly higher than that in CCC-N (Figure 3B, *P* < 0.01). Double immunostaining for MCM2 and CC3 demonstrated the exclusive expression of MCM2 or CC3 in the nuclei of CCC-N cases (Supplementary Figure 3A), whereas cytoplasmic MCM2 and nuclear CC3 were co-expressed in CCC-C cells (Supplementary Figure 3B).

### Transfection of MCM2-ΔN into OVTOKO and OVISE cells

**Figure 4:**
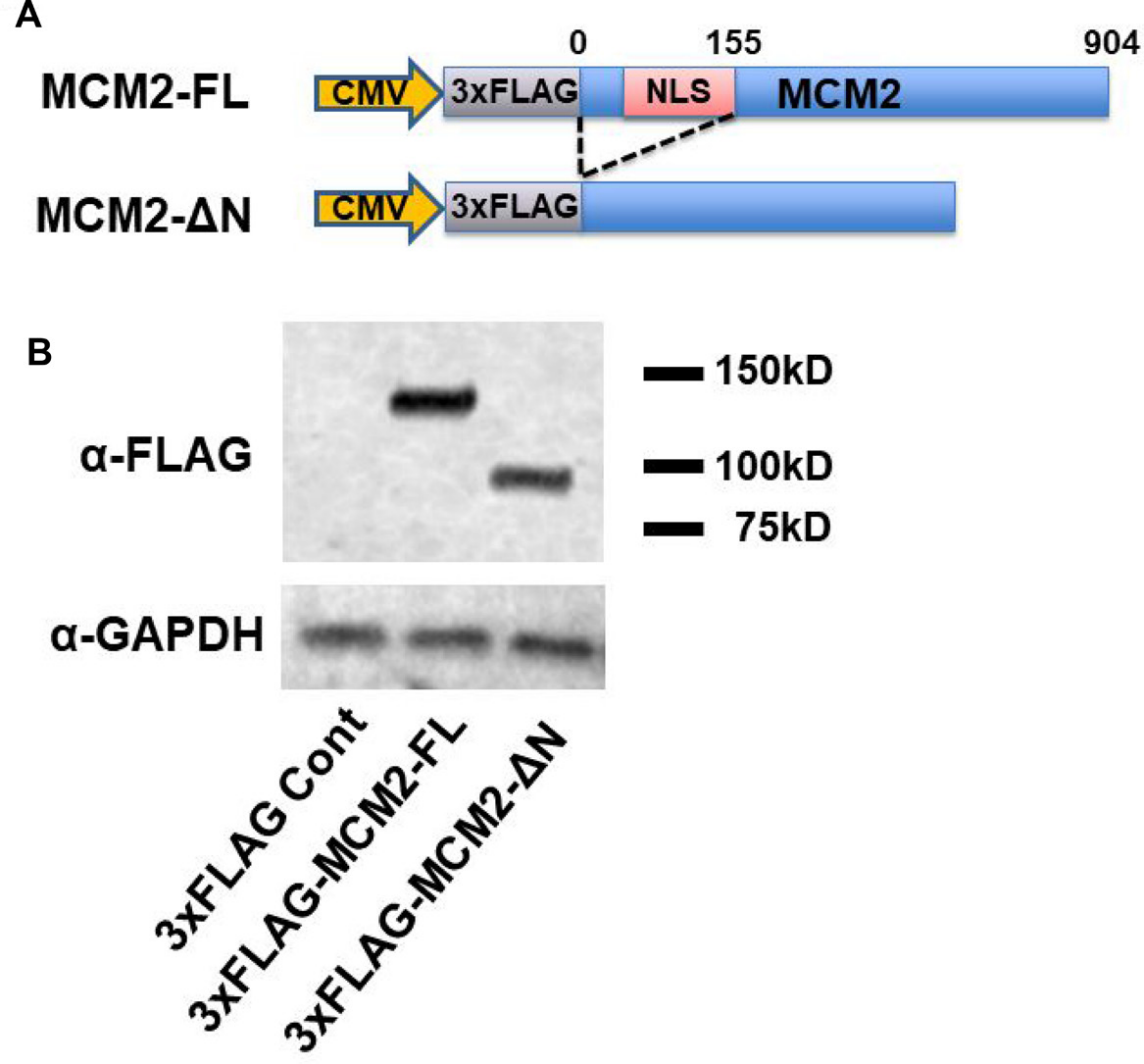

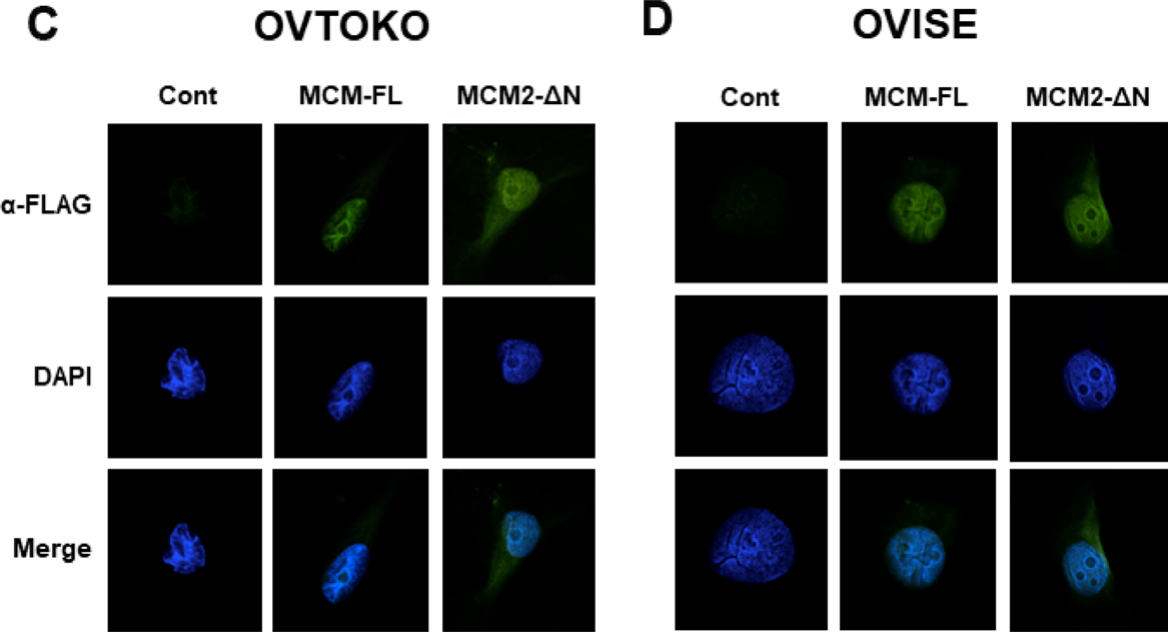
Transfection of *3*×*FLAG-MCM2-ΔN* that lacks the NLS domain of minichromosome maintenance 2 (MCM2) resulted in the cytoplasmic expression of MCM2 in OVTOKO and OVISE cells (**A**) Schematic diagram of full-length MCM2 (MCM2-FL) and MCM2 deletion mutants [MCM2-ΔN (aa 156–904)]. (**B**) Western blot analysis to confirm that both 3×FLAG-MCM2-FL and 3×FLAG-MCM2-ΔN were expressed as the appropriate-size protein. (**C**, **D**) Subcellular localization of transduced MCM-FL and MCM-ΔN in OBTOKO and OVISE cells was demonstrated by fluorescent immunostaining using an anti-FLAG antibody. MCM-FL was localized to the nucleus, while MCM2-ΔN was expressed in both cytoplasm and nucleus of OVTOKO (**C**) and OVISE (**D**) cells.

As shown in Figure 4A, *MCM2-*Δ*N* was generated to transduce the MCM2-ΔN protein, which lacks the NLS domain of MCM2. To confirm successful transfection, western blot analysis was performed to determine the appropriate size of transduced MCM2-FL (full length) and MCM2-ΔN proteins using an anti-FLAG antibody (Figure 4B). Furthermore, subcellular localization of transduced proteins was demonstrated by fluorescence immunostaining using an anti-FLAG antibody. MCM-FL was localized to the nucleus, whereas MCM2-ΔN was expressed in both cytoplasm and nucleus of OVTOKO (Figure 4C) and OVISE (Figure 4D) cells. The intrinsic MCM2 protein was localized to the nucleus both in OVTOKO and OVISE cells as determined by immunostaining using the anti-MCM2 antibody (Supplementary Figure 4A and 4B).

To observe the effects of cell proliferation on the expression of MCM2-FL and MCM2-ΔN, cell numbers were calculated. Each cell did not exhibit significant difference in number for 24, 48 and 72 h in OVTOKO and OVISE cells. (Supplementary Figure 5)

**Figure 5:**
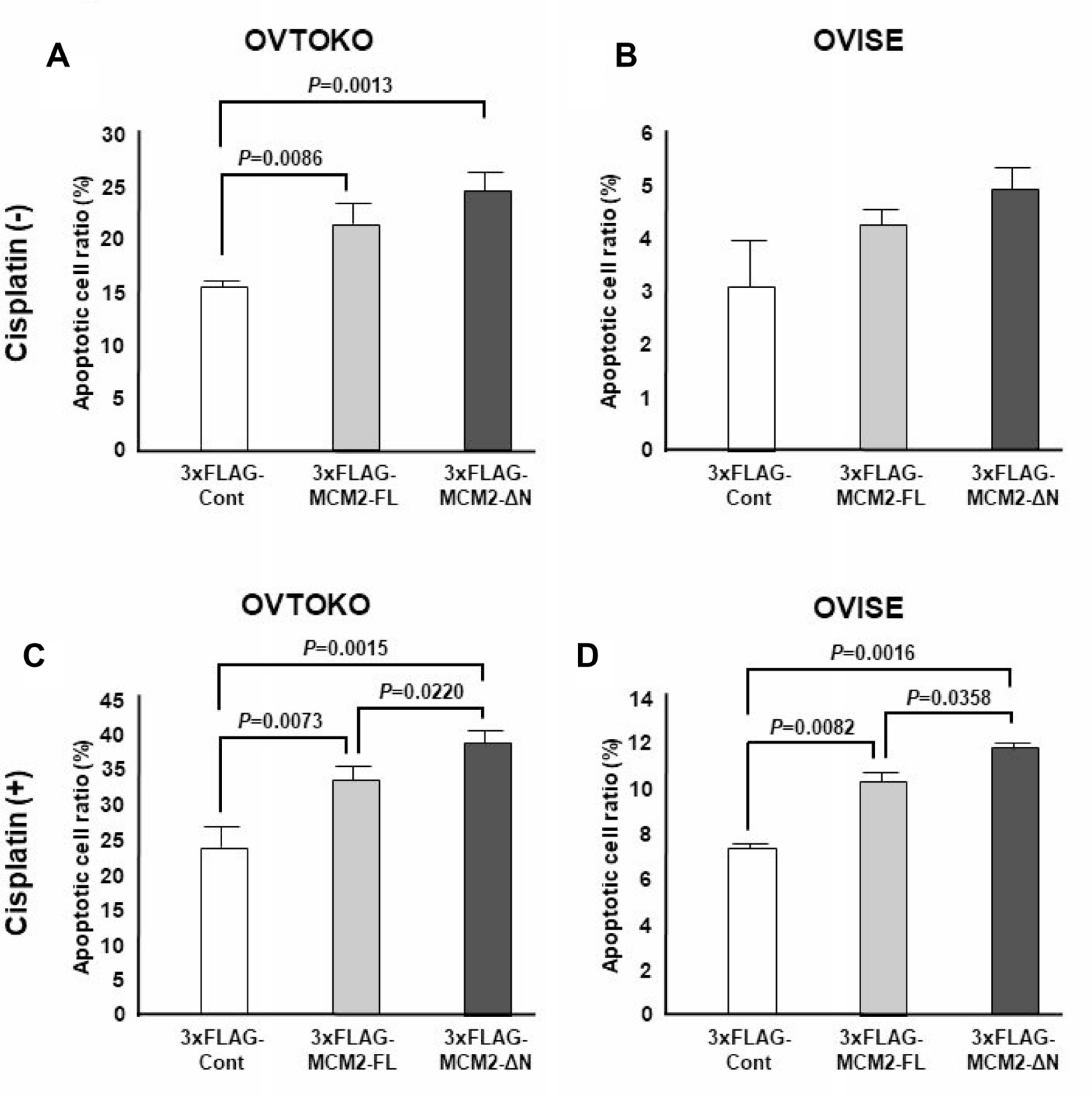
The apoptotic cell ratio induced by cisplatin treatment in full-length minichromosome maintenance 2 (MCM2-FL)- and NLS domain-deficient MCM2 (MCM2-ΔN)-transduced cells was determined by FACS analysis after annexin V staining *P* values were calculated using Student's *t*-test. The annexin V-positive cell ratio was assessed in control and MCM2-FL- and MCM2-ΔN-transduced OVTOKO and OVISE cells with or without cisplatin treatment. The transfection of MCM2-FL and MCM2-ΔN resulted in a significant increase of the apoptotic cell ratio in OVTOKO cells compared with control cells. In contrast, MCM2-FL and MCM2-ΔN transfection did not cause a significant increase of apoptotic cells in OVISE cells. After treatment with cisplatin, cells with MCM2-ΔN transduction exhibited significantly stronger induction of apoptosis than MCM-FL-transduced OVTOKO and OVISE cells did. Each experiment was performed three times.

To determine whether cytoplasmic localization of MCM2 influenced apoptotic induction by cisplatin in OVTOKO and OVISE cells, the ratio of annexin V-positive cells was assessed in control and MCM2-FL- and MCM2-ΔN-transduced OVTOKO and OVISE cells with or without cisplatin treatment. As shown in Figure 5, transduction of MCM2-FL and MCM2-ΔN resulted in a significant increase of the apoptotic cell ratio in OVTOKO cells compared with that in control cells (Figure 5A). In contrast, MCM2-FL and MCM2-ΔN transfection did not induce a significant increase in apoptotic OVISE cells (Figure 5B). However, after treatment with cisplatin, both cell types revealed significant induction of apoptosis with MCM2-FL transfection (Figure 5C, 5D). Furthermore, cells with MCM2-ΔN transfection exhibited a significantly higher rate of apoptosis than that in MCM-FL-transduced OVTOKO and OVISE cells (Figure 5C, 5D). These results suggest that cytoplasmic localization of the MCM2 protein significantly enhanced cisplatin-induced apoptosis in human ovarian cancer cell lines.

## DISCUSSION

Patients with clear cell carcinoma are typically young and are often diagnosed with stage I–II disease. Therefore, they do not exhibit poor prognosis [21]. In the present study, the majority of clear cell carcinoma cases presented as stage I or II (46 out of 60 cases) with a 5-year overall survival rate of approximately 80%. However, patients with higher stages of clear cell carcinoma have poorer prognosis than those with serous carcinoma [22, 23]. Clear cell carcinoma patients with stage IC disease or higher have greater risk of recurrence [24]. In this study, among the stage IC patients, CCC-N and CCC-C cases accounted for 35.7% (5/14) and 10% (1/10) of the AWD and DOD individuals, respectively. This result suggests that cytoplasmic expression of MCM2 may correlate with CCC chemo-sensitivity. Advanced disease is associated with very poor prognosis and resistance to standard treatments [25].

To predict the prognostic features of clear cell carcinoma of the ovary, several markers have been investigated by immunohistochemistry. For example, the Ki-67 labeling index facilitates the diagnostic differentiation of ovarian carcinoma subtypes, thereby predicting prognosis and determining the need for chemotherapy [26]. Both Ki-67 and MCM2 are proliferation markers. In this study, there was not significant correlation between the labelling indices of MCM2 and Ki-67, suggesting that MCM2 may contribute to human ovarian cancer with a function different from proliferation. Overexpression of glypican-3 may be related to low-level proliferation of tumor cells and is associated with resistance to chemotherapy and poor prognosis of clear cell carcinoma [27]. Furthermore, the expression of EMI1 protein in clear cell carcinoma is correlated with high histologic grade and worse overall survival [28]. Regarding the expression of MCM2 protein in ovarian cancers, the immunohistochemical expression in ovarian high-grade serous carcinomas was higher than that in endometrioid carcinomas [29]. The frequency of Ki-67, MCM2, geminin, and aurora A and B was significantly associated with tumor grade and ploidy status in epithelial ovarian carcinomas [30]. Similarly, the labeling indices of MCM2 and MCM5 proteins of ovarian adenocarcinomas were associated with adverse patient outcomes in univariate and multivariate analyses. Therefore, MCM2 and MCM5 proteins are promising prognostic markers in patients with ovarian adenocarcinoma [16].

Because adjuvant chemotherapy had little impact on the survival of stage I clear cell carcinoma patients [31], further study is needed to improve the survival rate associated with this disease. Platinum-based chemotherapy is one of the most effectively employed chemotherapeutic treatments for cancers including ovarian cancer. The mechanism of action of platinum-based compounds is related to DNA damage-induced apoptosis [32]. The most commonly used platinum-containing agents are cisplatin, its analog carboplatin, and oxaliplatin. The majority of ovarian cancer patients are treated with platinum-based chemotherapy; however, the resistance to such chemotherapy drugs severely limits their overall efficacy [33]. Although patients with serous histological tumors were relatively sensitive to platinum-based chemotherapy, clear cell carcinoma and mucinous carcinoma demonstrate a chemo-resistant phenotype leading to poorer prognosis [1].

We have previously shown that cytoplasmic localization of MCM2 enhanced DNA damage-induced apoptosis in hematopoietic cells [20]. Here, we demonstrated the cytoplasmic expression of MCM2 in a subset of ovarian cancer patients with clear cell carcinoma. In these specific patients, DNA damage-induced apoptosis was markedly enhanced during the course of platinum-based chemotherapy after surgical operation. Even at the time of operation when the surgical samples were removed, these tumor cells exhibited high frequency of apoptosis in the tumor, indicating high susceptibility to apoptotic stimulation such as reactive oxygen species production or ischemia. These cases demonstrate the possible remarkable outcome of effective chemotherapy using platinum-based agents. In addition, cytoplasmic MCM2 expression provides a key indicator of the effectiveness of MCM2-targeted therapy against clear cell carcinoma in the future.

## MATERIALS AND METHODS

### Patients and pathological specimens

We examined pathological specimens obtained from 174 ovarian epithelial cancer patients (60 serous carcinoma, 37 endometrioid carcinoma, and 77 clear cell carcinoma) at Tokyo Medical and Dental University Hospital, Tokyo, between 1998 and 2016. Pathological diagnosis was confirmed according to the WHO criteria by two pathologists (K.Y. and M.K.). Specimens were obtained by surgical resection, routinely fixed in 10% neutralized formalin, and embedded in paraffin for conventional histopathological examination. We obtained informed consent from all patients. Furthermore, this study was approved by the ethics committees of Tokyo Medical and Dental University, and all procedures were performed in accordance with the ethical standards established by these committees (M2000–1458).

### Immunohistochemistry

Formalin-fixed, paraffin-embedded (FFPE) tissue was sliced (4-μm thickness), and the sections were placed on silane-coated slides. Post deparaffinization, heat-based antigen retrieval at 95° C for 20 min in citrate buffer (pH 6.2), endogenous peroxidase blockade using 3% hydrogen peroxide, and blocking with normal horse serum (ABC Kit; Vector Laboratories, Burlingame, CA, USA) were performed. The primary antibodies used were as follows: anti-MCM2 (BM28; targeted to the C-terminus); mouse monoclonal, clone no. 610701, 1:2,000 (BD Transduction Laboratories, Cambridge, MD, USA); anti-MCM2 (N-19; targeted to the N-terminus); goat polyclonal, clone no. sc-9839, 1:250 (Santa Cruz Biotechnology, Inc., Dallas, TX, USA); anti-Ki-67 (MIB-1), mouse monoclonal, clone no. M7240, 1:200 (DAKO, Tokyo, Japan); and anti-cleaved Caspase-3 (CC3) (Asp175), rabbit monoclonal, clone no. 5A1E, 1:250 (Cell Signaling Technology, Danvers, MA, USA). Specimens treated with primary antibodies were incubated overnight at 4° C. Primary antibodies were detected using the ABC Kit (Vector Laboratories) for MCM2 (BM28), Histofine Simple Stain™ (Nichirei Bioscience, Tokyo, Japan) for MCM2 (N-19) and CC3, and diaminobenzidine (DAB; Vector Laboratories). Counter staining was performed using hematoxylin. The positivity of each protein expression was calculated in 10 areas of high-power field randomly chosen.

### Double immunostaining

For double immunostaining of MCM2 (BM28) and CC3, FFPE tissue sections (4-μm thickness) were deparaffinized and pre-treated by microwave unmasking at 95° C for 20 min in citrate buffer (pH 6.2). After inhibition of endogenous peroxidase activity and non-specific protein binding as described in the previous section, tissue sections were incubated with primary antibody [dilution 1:2,000 for MCM2 (BM28)] overnight at 4° C. The ABC Kit and DAB as the chromogen were used for antibody detection. Then, sections were pretreated with microwave unmasking at 95° C for 20 min in citrate buffer (pH 6.2). Non-specific protein binding was blocked by incubation with 10% normal goat serum. Next, sections were incubated with antibodies against CC3 (1:250, overnight at 4° C). Detection was performed using a secondary antibody of the Histofine Simple Stain AP (M) system (Nichirei Bioscience) with Vector Blue (Vector Laboratories) as the chromogen for CC3. Counter staining was performed using hematoxylin.

### Evaluation of immunohistochemistry

To evaluate the labeling indices (LIs) of MCM2 (BM28), MCM2 (N19), Ki-67, and CC3, positively stained cells were counted in 10 areas of high-power field chosen randomly. The LIs were expressed as the percentage of positively stained cells based on a count of at least 1,000 cancer cells.

### Cell lines and culture

The OVTOKO and OVISE cell lines were purchased from the Japanese Collection of Research Bioresources (Tokyo, Japan). Both OVTOKO and OVISE cells originated from clear cell carcinoma tissue. The cells were cultured in Dulbecco's Modified Eagle's Medium (DMEM) supplemented with L-glutamine, phenol red (Wako Pure Chemical Industries, Ltd., Osaka, Japan), 10% fetal bovine serum (FBS), and 1% penicillin-streptomycin solution. Cells were cultured at 37° C with 5% CO_2_. Cells were passaged at a ratio of 1:5–1:8 every 2–3 days.

### Establishment of 3×FLAG-tagged *MCM2*-overexpressing vector

Human 3×FLAG-tagged full-length *MCM2* (*3*×*FLAG-MCM2-FL*) and 3×FLAG-tagged N-terminal-deficient *MCM2* (*3*×*FLAG-MCM2-*ΔN) were subcloned using the cDNA from HEK293T cells. PCR primers for *MCM2-FL* used in this study have been previously described [21]. The PCR primers for *MCM2-ΔN* were as follows: forward primer: 5′-AATATGCGGCCGCGCG GGCCACGGAGGACGGCG-3′ and reverse primer: 5′-AGCGGCCGCAAGCAGGCTTGGAGAAACAA-3′. PCR products were inserted into the *p3*×*FLAG-CMV™-10* Expression Vector (Sigma, St. Louis, MO, USA). The protein levels of *3*×*FLAG-MCM2-FL* and *3*×*FLAG-MCM2-ΔN* were confirmed by western blotting as previously described [34].

### Transfection of *3*×*FLAG-tagged MCM2-FL* and *MCM2-*ΔN

The *3*×*FLAG-MCM2-FL* and its construct, *3*×*FLAG-MCM2-*ΔN, were transfected into OVTOKO and OVISE cells (1 × 10^6^ cells) using the Amaxa^®^ Cell Line Nucleofector^®^ Kit V (Program No. X-001, Lonza, Basel, Switzerland). The control subjects were generated by sham treatment using an empty vector in each cell line.

### Immunofluorescent microscopy for subcellular localization of 3×FLAG-MCM2-FL and 3×FLAG-MCM2-ΔN

Ten thousand OVTOKO and OVISE cells, which overexpressed FLAG-MCM2-FL and 3×FLAG-MCM2-ΔN, were cultured on Falcon^®^ 4 Well Culture Slides (BD Falcon NJ, USA). Cells were fixed in 100% ethanol at −20° C for 20 min and then incubated with rabbit monoclonal anti-FLAG antibody (Sigma) at a 1:100 dilution in PBS for 1 h at room temperature. Then, they were stained with a TRITC-conjugated anti-rabbit antibody (Dako Cytomation, Glostrup, Denmark) at a 1:100 dilution for 20 min at room temperature. Slides were washed three times with PBS and mounted with mounting medium (Dako Cytomation) containing 4′,6-diamidino-2-phenylindole (DAPI, Abbott Molecular Inc., Des Plaines, IL, USA). Images were acquired using a FV1200 laser-scanning microscope (OLYMPUS, Tokyo, Japan) with a 1,000× objective.

### Assessment of cell number and viability in OVTOKO and OVISE cells with 3×FLAG-MCM2-FL and 3×FLAG- MCM2-ΔN overexpression

Cell number and viability upon overexpression of 3×FLAG- MCM2-FL and 3×FLAG- MCM2-ΔN were analyzed and compared with those of control cells. The cells were prepared at a density of 1 × 10^6^. The *3×FLAG-MCM2-FL*, *3×FLAG-MCM2-*ΔN, *and* control vector were transfected into OVTOKO and OVISE cells, which were then seeded in 24-well plates. After 24 h, OVTOKO and OVISE cells were treated with 1 μM and 5 μM cisplatin (WAKO, Tokyo, Japan), respectively. To determine each cell number at 24, 48, and 72 h, Countess® II FL (Thermo Fisher Scientific K. K., Tokyo, Japan) was used. After 24 h, cells were harvested, stained with a Cy5-labeled anti-annexin V antibody (BD Bioscience, San Jose, CA, USA), and analyzed by flow cytometry using a BD FACSCanto™ II analyzer (Becton Dickinson and Company, Franklin Lakes, NJ, USA).

### Statistical analysis

Correlations between two groups were examined by Student's *t*-test, Fisher's exact test, Pearson's correlation efficiency, and Spearman's correlation coefficient. Correlation analysis of overall survival was performed from the date of diagnosis to the date of last follow-up or death. Kaplan-Meier survival curves were used to estimate overall survival rates, and the log-rank test was used to assess differences in survival between groups. Univariate and multivariate analyses were performed using the Cox proportional hazard regression model. All differences with *P* ≤ 0.05 were considered statistically significant. Ekuseru-Toukei 2012 version 1.15 (Social Survey Research Information Co., Ltd, Tokyo, Japan) was used for all analyses.

## SUPPLEMENTARY MATERIALS FIGURES


