# Supplementary material for: Subcellular localization of MCM2 correlates with the prognosis of ovarian clear cell carcinoma
Source: Oncotarget. 2018 Jun 15;9(46):28213–25. doi: 10.18632/oncotarget.25613 (PMC6021330; doi:10.18632/oncotarget.25613)
Supplement: Supplementary file 1 [file oncotarget-09-28213-s001.pdf]

Subcellular localization of MCM2 correlates with the prognosis of ovarian clear cell carcinoma

SUPPLEMENTARY MATERIALS

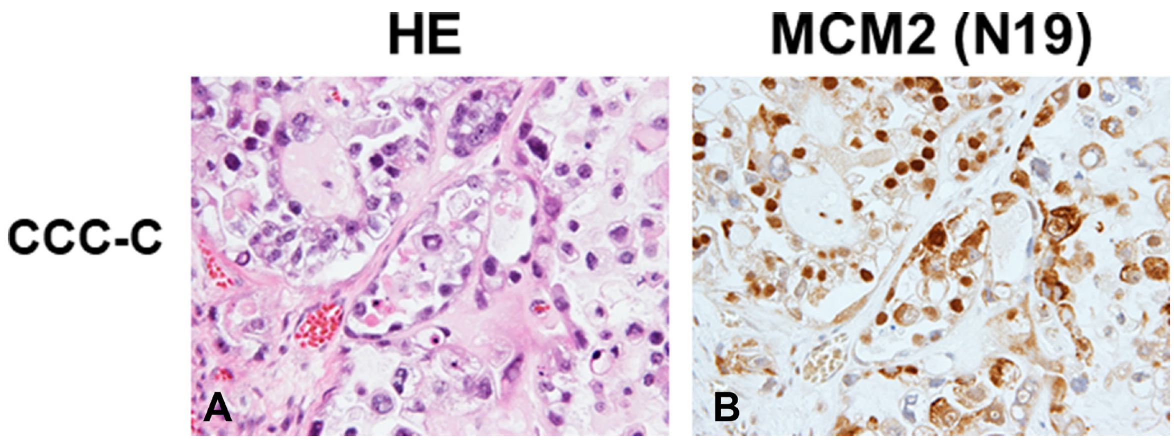

Supplementary Figure 1: The staining patterns of minichromosome maintenance 2 (MCM2) expression were similar in clear cell carcinoma with cytoplasmic/nuclear expression of MCM2 (CCC-C) cases when using the N19 and BM28 antibodies. [(**A**) hematoxylin and eosin staining, (**B**) MCM2 (N19)].

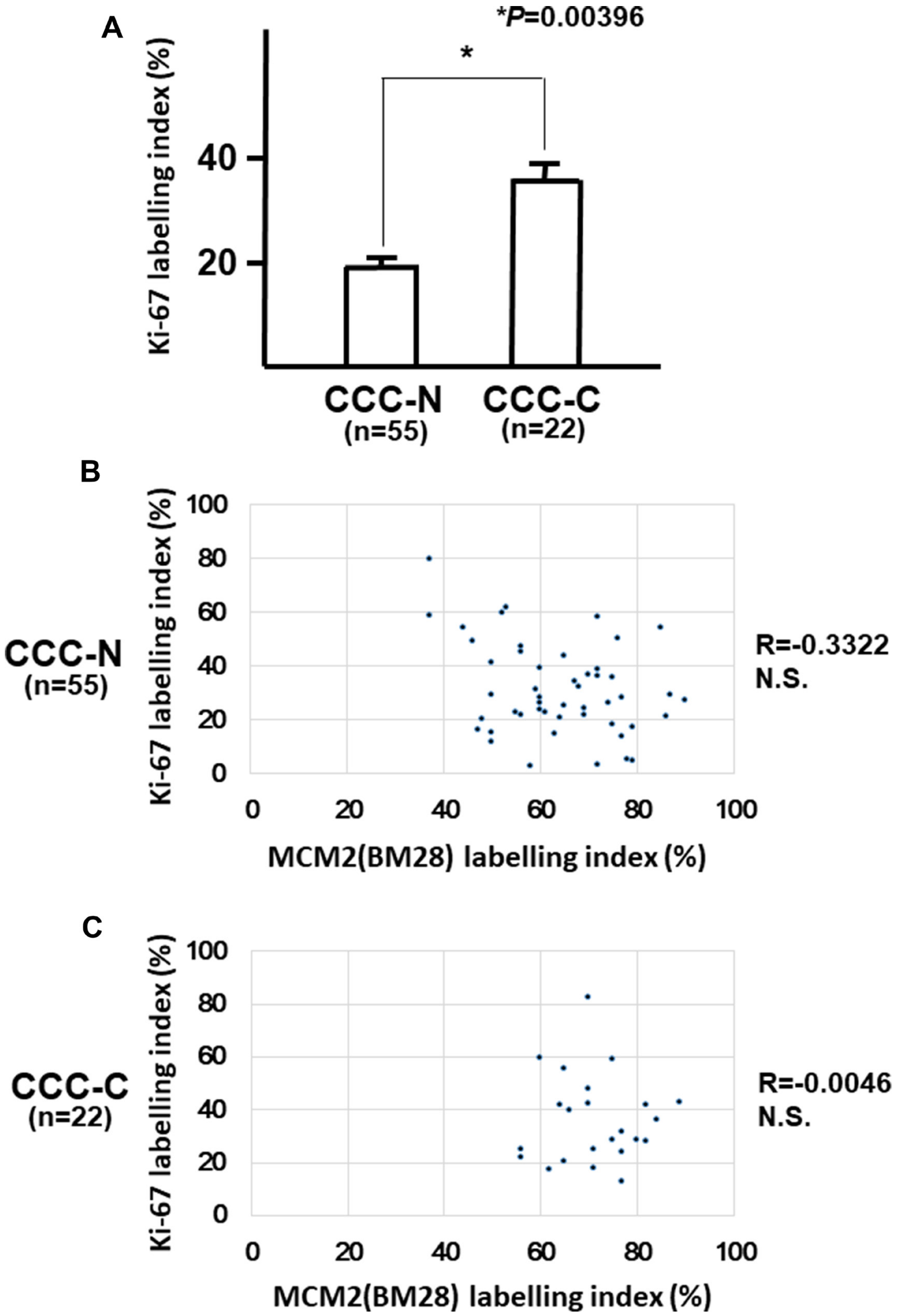

Supplementary Figure 2: (**A**) Positive ratio of Ki-67 in clear cell carcinoma with cytoplasmic/nuclear expression of minichromosome maintenance 2 (MCM2; CCC-N) and clear cell carcinoma with nuclear expression of MCM2 (CCC-C). Ki-67 positive ratio in CCC-C was significantly higher than that in CCC-N. *P* values were calculated using Student's *t*-test. (**B**) The correlation of positive ratio between MCM2 (BM28) and Ki-67. Each positivity was not statistically correlated (CCC-N; $R = -0.3322$, CCC-C; $R = -0.0046$).

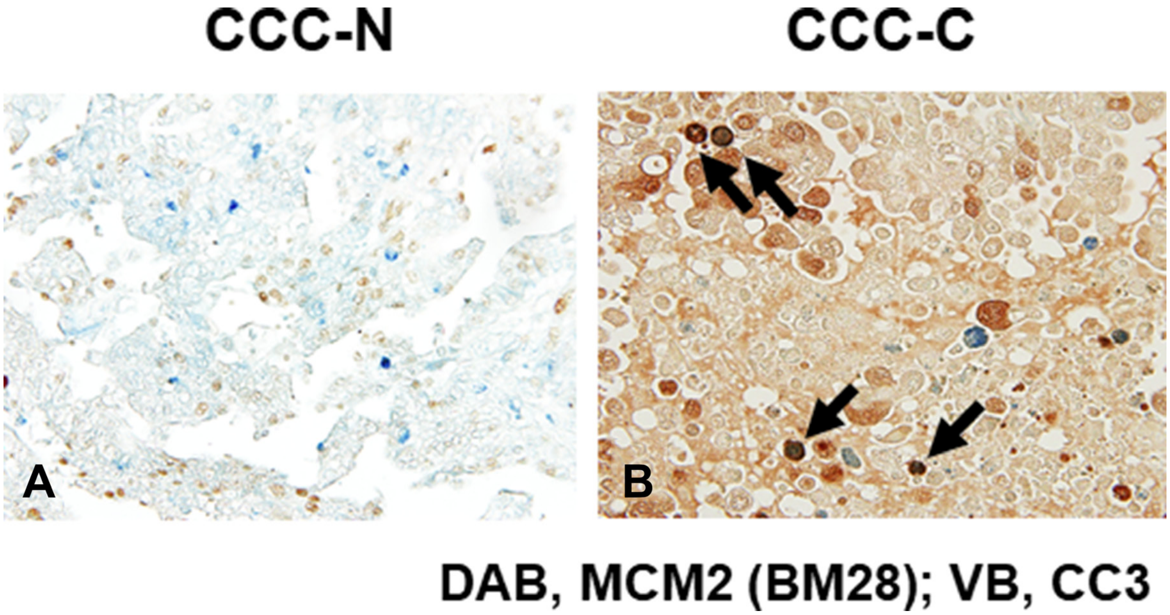

Supplementary Figure 3: Double immunostaining for minichromosome maintenance 2 [MCM2; developed with 3-3′-diaminobenzidine (DAB), brown] and CC3 (developed with Vector Blue (VB), blue) demonstrated the exclusive expression of MCM2 and CC3 in the nuclei of clear cell carcinoma with nuclear expression of MCM2 (CCC-N) cases (**A**), whereas cytoplasmic MCM2 and nuclear CC3 were co-expressed in clear cell carcinoma with nuclear expression of MCM2 (CCC-C) cells (**B**).

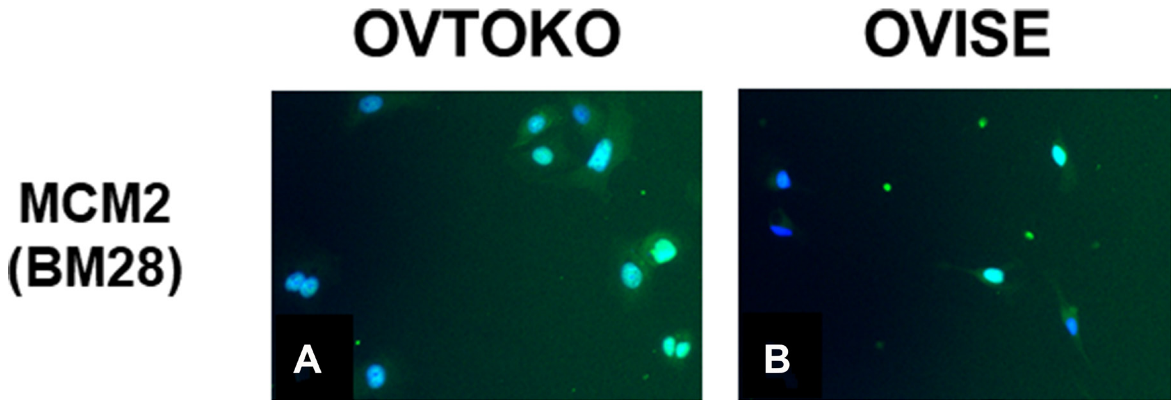

Supplementary Figure 4: Subcellular localization of endogenous minichromosome maintenance 2 (MCM2) protein. Endogenous MCM2 protein is localized in the nucleus in OVTOKO (**A**) and OVISE cells (**B**).

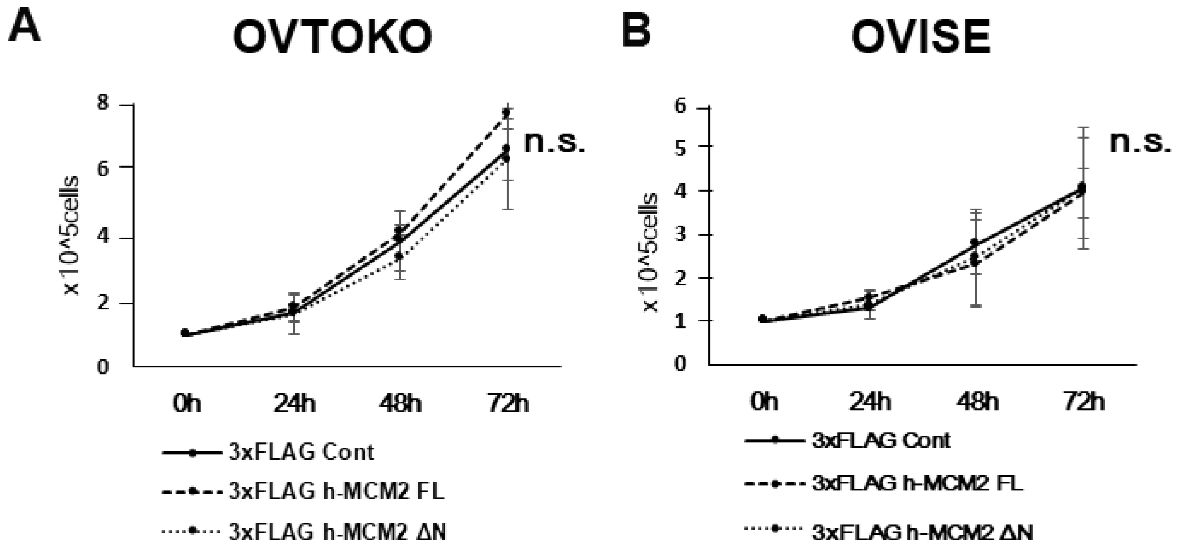

Supplementary Figure 5: Cell proliferation analysis under the expression of MCM2-FL and MCM2-ΔN. Each cell did not exhibit significant difference in number for 24, 48 and 72 h in OVTOKO (**A**) and OVISE cells (**B**). MCM2: minichromosome maintenance 2; MCM2- ΔN: MCM2 lacking the NLS domain, N.S.: not significant.
